# Dataset of rice growth for saline-alkaline tolerance screening

**DOI:** 10.1016/j.dib.2025.111998

**Published:** 2025-08-18

**Authors:** Mami Nampei, Akihiro Ueda

**Affiliations:** Graduate School of Integrated Sciences for Life, Hiroshima University, Higashi-Hiroshima, Hiroshima, 739-8528, Japan

**Keywords:** Growth, Rice, Rice core collections, Saline-alkaline stress, Screening

## Abstract

This dataset exhibits the growth profile of multiple rice varieties, most of which include world or Japanese rice core collections, under saline-alkaline conditions through two screenings. In both the first and second screenings, the rice plants were hydroponically cultivated for 4 weeks under normal conditions and then subjected to control or saline-alkaline conditions for 2 weeks. In the first screening, dry weight, dry weight ratio, and SPAD values were measured, and candidate varieties possessing saline-alkaline tolerance (7 varieties) or sensitivity (3 varieties) were selected based on the dry weight ratio. In the second screening, the fresh/dry weight, fresh/dry weight ratio, length of roots and shoots, and chlorophylls were measured in totally 12 rice varieties. These include the 7 tolerant and 3 sensitive varieties identified in the first screening, as well as 1 known saline-tolerant and 1 saline-sensitive variety. The dataset is expected to be useful for understanding the differences in saline-alkaline tolerance in multiple rice varieties and for breeding new saline-alkaline tolerance rice cultivars.

Specifications Table

**Table:** 

Subject	Biology
Specific subject area	Growth profile of rice varieties under saline-alkaline conditions
Type of data	Table, Graph. Raw.
Data collection	Rice plants were cultivated in a hydroponic solution containing half-length modified Kimura B solution in a greenhouse for 4 weeks [1,2]. The seedlings were then exposed to control, saline-alkaline (SA)-pH 8.3, and SA-pH 8.7 (only in the first screening) conditions for 2 weeks. In the first screening, the measured parameters include dry weight, dry weight ratio, and SPAD values. The second screening included fresh/dry weight, dry weight ratio, length of roots and shoots, and chlorophyll a, b, and *a* + *b*. The experiment was conducted with four replicates, each consisting of one individual rice plant.
Data source location	Institution: Hiroshima University, Location: Kagamiyama, Higashi-Hiroshima, Hiroshima, Japan Latitude and longitude: 34.397° N, 132.717° E
Data accessibility	Repository name: Mendeley Data Data identification number: 10.17632/mj5g26f36t.2 Direct URL to data: https://data.mendeley.com/datasets/mj5g26f36t/2
Related research article	M. Nampei, H. Ogi, T. Sreewongchai, S. Nishida, A. Ueda, Potassium transporter OsHAK17 may contribute to saline-alkaline tolerant mechanisms in rice (Oryza sativa), J. Plant Res. 137 (2024) 505–520. https://doi.org/10.1007/s10265–024–01,529–0.

## 1 Value of the Data

• This is the first study to evaluate the saline-alkaline tolerance of rice varieties included in the world or Japanese rice core collections. The data in this study are useful because they show the effects of saline-alkaline stress on the growth of 35 rice varieties. Physiological and molecular studies can be conducted to understand the mechanisms of saline-alkaline stress tolerance and to identify tolerant genes or loci in rice using the selected rice varieties and data presented in this article.
• The rice varieties selected as saline-alkaline tolerant are expected to become the parental lines of new tolerant rice cultivars in the future.
• The multiple saline-alkaline tolerant rice candidates identified here can be re-evaluated to identify regionally adapted breeding materials for the development of saline-alkaline tolerant rice cultivars.

## 2 Background

Rice is an important staple food that provides energy for humans, particularly in Asian countries. However, rice production is severely affected by salt-affected soils in arid and semiarid areas worldwide. In particular, rice cropping in saline-alkaline soils, which contain alkaline salts such as NaHCO_3_ and Na_2_CO_3_, is extremely challenging compared to that in saline soils at neutral pH [3]. Saline-alkaline stress is a complex stress consisting of osmotic, ionic, oxidative, and alkaline components [4]. Together, these factors can cause severe damage to plants. Plants show multiple symptoms, including dehydration, chlorosis, Na^+^ overaccumulation, nutrient deficiency, and restricted growth, ultimately leading to death [4]. Thus, exploring saline-alkaline-tolerant rice varieties, understanding saline-alkaline tolerance mechanisms, and generating new tolerant rice cultivars are critically needed.

In a previous study, screening and breeding of salt-tolerant rice varieties were conducted, resulting in the development of cultivars that perform well under salt stress at neutral pH levels [5,6]. However, these tolerant varieties may not necessarily possess saline-alkaline tolerance, as it remains unclear whether their tolerance expands to saline conditions at high pH levels [7]. In this study, we screened rice varieties under saline-alkaline conditions to identify genotypes useful for understanding saline-alkaline tolerance mechanisms and breeding new saline-alkaline tolerant cultivars.

## 3 Data Description

**Fig. 1:**
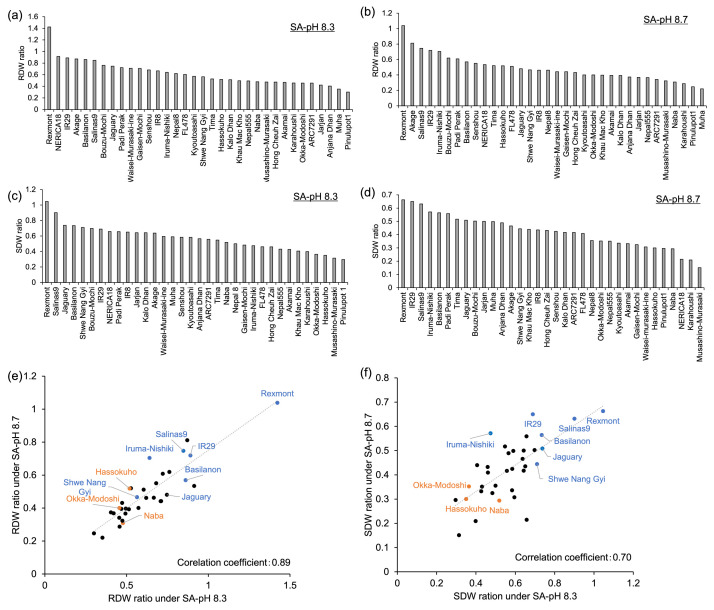
(a-d) Dry weight ratio of the roots (a, b) and shoots (c, d) under SA-pH 8.3 (a, c) and SA-pH 8.7 (b, d). (e, f) Scatter plots showing root dry weight (RDW) ratio (e) and shoot dry weight (SDW) ratio (f) under SA-pH 8.3 (50 mM NaHCO_3_) and SA-pH 8.7 (47.5 mM NaHCO_3_ + 1.25 mM Na_2_CO_3_). The ratio was calculated as the dry weights under SA-pH 8.3 or SA-pH 8.7 conditions related to those under control conditions in the first screening. Blue and yellow colors indicate SA-tolerant and -sensitive varieties selected in the first screening, respectively.

**Fig. 2:**
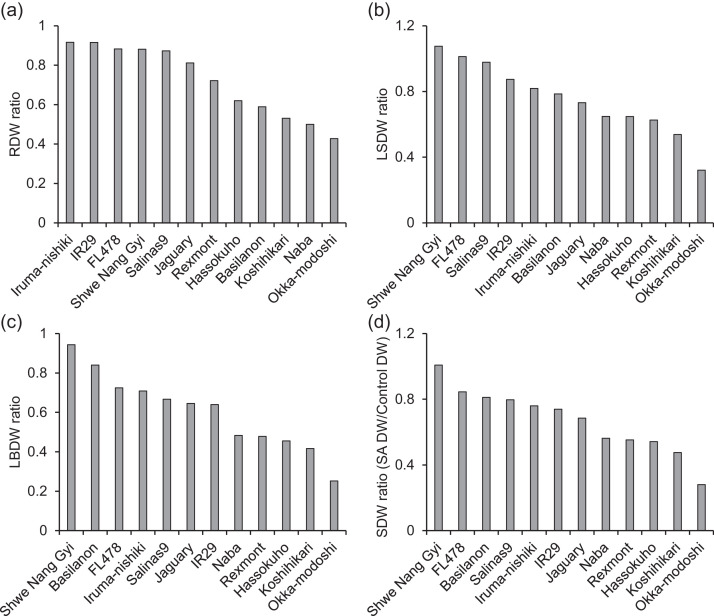
Dry weight ratio of the roots (a), LS (b), LB (c), and shoots (LB+LS) (d) under SA-pH 8.3 (50 mM NaHCO_3_). The ratio was calculated as the dry weights under SA-pH 8.3 conditions related to those under control conditions in the second screening.

The dataset in this article presents the growth results of various rice varieties with saline-alkaline tolerance or sensitivity. All data in this article are available in [8] Data were collected over two screening cycles using 35 rice genotypes (Table 1). In the first screening, the dry weights of the roots and shoots (Table 2, 3) and SPAD (Soil and Plant Analyzer Development) values (Table 4) were measured. Additionally, the shoot dry weight ratio and correlation coefficient of root and shoot dry weight ratio under SA-pH 8.3 and SA-pH 8.7 were calculated (Fig. 1). In the second screening, the SA-tolerant/sensitive rice varieties selected from the first screening based on the shoot dry weight ratio, FL478 (index of salt-tolerant rice), and Koshihikari (index of salt-sensitive rice) were used. Measurements included the length of the roots and shoots (Table 5), fresh and dry weights of the roots, leaf sheath (LS), leaf blades (LB) (Table 6, Table 7), and chlorophyll a, b, and total chlorophyll *a* + *b* (Table 8). The dry weight ratios of the roots, LS, LB, and shoots (LS + LB) were calculated based on the dry weight of each organ (Fig. 2).

**Table 1:** List of rice varieties as materials. The symbols in the ``Selected varieties for 2nd screening'' column have the following meanings. 〇: Candidates of saline-alkaline tolerant rice varieties. ●: Candidates of saline-alkaline sensitive rice varieties. △: A salt-tolerant rice variety that is set as an index in the second screening. ▲: A salt-sensitive rice variety that is set as the index in the second screening.

No.	Name	Subspecies	type	Origin	Varieties for 2nd screening
**1**	**Naba**	**Indica**	**Landrace**	**India**	**●**
**2**	**Shwe Nang Gyi**	**Indica**	**Landrace**	**Myanmar**	**〇**
**3**	**Pinulupot1**	**Indica**	**Landrace**	**Philippines**	
**4**	**Muha**	**Indica**	**Landrace**	**India**	
**5**	**Nepal8**	**Indica**	**Landrace**	**Nepal**	
**6**	**Jarjan**	**Indica**	**Landrace**	**Bhutan**	
**7**	**Kalo Dhan**	**Indica**	**Landrace**	**Nepal**	
**8**	**Anjana Dhan**	**Indica**	**Landrace**	**Nepal**	
**9**	**ARC7291**	**Indica**	**Landrace**	**India**	
**10**	**Nepal555**	**Indica**	**–**	**India**	
**11**	**Basilanon**	**Indica**	**Landrace**	**Philippines**	**〇**
**12**	**Jaguary**	**Tropical Japonica**	**–**	**Brazil**	**〇**
**13**	**Khau Mac Kho**	**Tropical Japonica**	**Landrace**	**Vietnam**	
**14**	**Padi Perak**	**Tropical Japonica**	**Landrace**	**Indonesia**	
**15**	**Rexmont**	**Tropical Japonica**	**Breeding**	**USA**	**〇**
**16**	**Tima**	**Tropical Japonica**	**Landrace**	**Bhutan**	
**17**	**Hong Cheuh Zai**	**Indica**	**–**	**China**	
**18**	**IR8**	**Indica**	**Breeding**	**Philippines**	
**19**	**NERICA18**	**–**	**Breeding**	**–**	
**20**	**Salinas9**	**Indica**	**Breeding**	**Philippines**	**〇**
**21**	**IR29**	**Indica**	**Breeding**	**Philippines**	**〇**
**22**	**FL478**	**Indica**	**Breeding**	**Philippines**	**△**
**23**	**Gaisen-Mochi**	**Tropical Japonica**	**Landrace**	**Japan**	
**24**	**Senshou**	**Tropical Japonica**	**Landrace**	**Japan**	
**25**	**Iruma-Nishiki**	**Tropical Japonica**	**Landrace**	**Japan**	**〇**
**26**	**Okka-Modoshi**	**Tropical Japonica**	**Landrace**	**Japan**	**●**
**27**	**Bouzu-Mochi**	**Tropical Japonica**	**Landrace**	**Japan**	
**28**	**Akage**	**Japonica**	**Landrace**	**Japan**	
**29**	**Hassokuho**	**Japonica**	**Landrace**	**Japan**	**●**
**30**	**Kyoutoasahi**	**Japonica**	**Landrace**	**Japan**	
**31**	**Akamai**	**Indica**	**Landrace**	**Japan**	
**32**	**Karahoushi**	**Indica**	**Landrace**	**Japan**	
**33**	**Waisei-Murasaki-Ine**	**–**	**–**	**Japan**	
**34**	**Musashino-Murasaki**	**–**	**–**	**Japan**	
**35**	**Koshihikari**	**Japonica**	**Breeding**	**Japan**	**▲**

**Table 2:** Dry weight of the roots (RDW) and shoots (SDW) under control, SA-pH 8.3 (pH 8.3: 50 mM NaHCO_3_), and SA-pH 8.7 (pH 8.7: 47.5 mM NaHCO_3_ + 1.25 mM Na_2_CO_3_) conditions. Data represent the means ± SE of four replicates. Different alphabets accompanying each value indicate statistically significant differences among treatments, as determined by one-way ANOVA followed by Tukey’s test (*p* < 0.05).

No.	Name	RDW (g)		
		Control	pH 8.3	pH 8.7
**1**	**Naba**	**0.34±0.06^a^**	**0.16±0.01^b^**	**0.11±0.02^b^**
**2**	**Shwe Nang Gyi**	**0.33±0.04^a^**	**0.19±0.01^b^**	**0.16±0.01^b^**
**3**	**Pinulupot1**	**0.61±0.18^a^**	**0.18±0.02^b^**	**0.15±0.03^b^**
**4**	**Muha**	**0.74±0.04^a^**	**0.26±0.04^b^**	**0.16±0.01^b^**
**5**	**Nepal8**	**0.40±0.04^a^**	**0.25±0.03^b^**	**0.18±0.01^b^**
**6**	**Jarjan**	**0.33±0.03^a^**	**0.14±0.01^b^**	**0.12±0.01^b^**
**7**	**Kalo Dhan**	**0.35±0.03^a^**	**0.18±0.01^b^**	**0.14±0.02^b^**
**8**	**Anjana Dhan**	**0.33±0.03^a^**	**0.13±0.01^b^**	**0.12±0.02^b^**
**9**	**ARC7291**	**0.31±0.05^a^**	**0.14±0.01^b^**	**0.11±0.01^b^**
**10**	**Nepal555**	**0.38±0.08^a^**	**0.19±0.01^b^**	**0.14±0.01^b^**
**11**	**Basilanon**	**0.16±0.00^a^**	**0.14±0.02^a^**	**0.09±0.01^b^**
**12**	**Jaguary**	**0.58±0.11^a^**	**0.44±0.04^ab^**	**0.28±0.02^b^**
**13**	**Khau Mac Kho**	**0.51±0.13^a^**	**0.25±0.02^ab^**	**0.20±0.01^b^**
**14**	**Padi Perak**	**0.24±0.04^a^**	**0.18±0.02^a^**	**0.15±0.01^a^**
**15**	**Rexmont**	**0.07±0.01^a^**	**0.09±0.02^a^**	**0.07±0.01^a^**
**16**	**Tima**	**0.37±0.03^a^**	**0.20±0.03^b^**	**0.19±0.03^b^**
**17**	**Hong Cheuh Zai**	**0.42±0.05^a^**	**0.20±0.01^b^**	**0.18±0.01^b^**
**18**	**IR 8**	**0.54±0.04^a^**	**0.36±0.01^b^**	**0.25±0.03^b^**
**19**	**NERICA18**	**0.15±0.01^a^**	**0.13±0.02^ab^**	**0.08±0.02^b^**
**20**	**Salinas9**	**0.20±0.02^a^**	**0.17±0.01^a^**	**0.15±0.02^a^**
**21**	**IR29**	**0.21±0.02^a^**	**0.19±0.02^a^**	**0.15±0.01^a^**
**22**	**FL478**	**0.84±0.08^a^**	**0.51±0.05^b^**	**0.43±0.04^b^**
**23**	**Gaisen-Mochi**	**0.60±0.09^a^**	**0.43±0.02^ab^**	**0.27±0.05^b^**
**24**	**Senshou**	**0.17±0.01^a^**	**0.12±0.00^b^**	**0.10±0.02^b^**
**25**	**Iruma-Nishiki**	**0.36±0.04^a^**	**0.23±0.01^b^**	**0.25±0.02^b^**
**26**	**Okka-Modoshi**	**0.31±0.02^a^**	**0.14±0.02^b^**	**0.13±0.02^b^**
**27**	**Bouzu-Mochi**	**0.21±0.01^a^**	**0.16±0.04^a^**	**0.13±0.02^a^**
**28**	**Akage**	**0.25±0.03^a^**	**0.22±0.03^a^**	**0.21±0.02^a^**
**29**	**Hassokuho**	**0.27±0.05^a^**	**0.14±0.02^b^**	**0.14±0.01^b^**
**30**	**Kyoutoasahi**	**0.48±0.08^a^**	**0.28±0.01^b^**	**0.19±0.03^b^**
**31**	**Akamai**	**0.55±0.04^a^**	**0.26±0.03^b^**	**0.22±0.01^b^**
**32**	**Karahoushi**	**0.62±0.05^a^**	**0.28±0.02^b^**	**0.18±0.01^b^**
**33**	**Waisei-Murasaki-Ine**	**0.13±0.01^a^**	**0.09±0.03^ab^**	**0.06±0.01^b^**
**34**	**Musashino-Murasaki**	**0.25±0.03^a^**	**0.12±0.01^b^**	**0.08±0.01^b^**

**Table 3:** Dry weight of the shoots (SDW) under control, SA-pH 8.3 (pH 8.3: 50 mM NaHCO_3_), and SA-pH 8.7 (pH 8.7: 47.5 mM NaHCO_3_ + 1.25 mM Na_2_CO_3_) conditions. Data represent the means ± SE of four replicates. Different alphabets accompanying each value indicate statistically significant differences among treatments, as determined by one-way ANOVA followed by Tukey’s test (*p* < 0.05).

No.	Name	SDW (g)		
		Control	pH 8.3	pH 8.7
**1**	**Naba**	**0.95±0.18^a^**	**0.49±0.05^b^**	**0.28±0.03^b^**
**2**	**Shwe Nang Gyi**	**0.99±0.10^a^**	**0.70±0.07^ab^**	**0.44±0.05^b^**
**3**	**Pinulupot1**	**1.91±0.56^a^**	**0.57±0.08^b^**	**0.57±0.10^b^**
**4**	**Muha**	**1.92±0.51^a^**	**1.13±0.13^a^**	**0.96±0.16^a^**
**5**	**Nepal8**	**1.36±0.02^a^**	**0.68±0.05^b^**	**0.48±0.03^c^**
**6**	**Jarjan**	**1.00±0.06^a^**	**0.64±0.04^b^**	**0.50±0.02^b^**
**7**	**Kalo Dhan**	**0.97±0.12^a^**	**0.62±0.05^b^**	**0.40±0.04^b^**
**8**	**Anjana Dhan**	**1.05±0.06^a^**	**0.60±0.04^b^**	**0.52±0.05^b^**
**9**	**ARC7291**	**1.05±0.14^a^**	**0.59±0.01^b^**	**0.44±0.05^b^**
**10**	**Nepal555**	**1.55±0.43^a^**	**0.67±0.01^ab^**	**0.54±0.07^b^**
**11**	**Basilanon**	**0.60±0.02^a^**	**0.44±0.03^b^**	**0.34±0.01^c^**
**12**	**Jaguary**	**1.79±0.32^a^**	**1.32±0.08^ab^**	**0.91±0.11^b^**
**13**	**Khau Mac Kho**	**1.62±0.35^a^**	**0.66±0.06^b^**	**0.71±0.02^b^**
**14**	**Padi Perak**	**0.77±0.10^a^**	**0.50±0.07^ab^**	**0.43±0.02^b^**
**15**	**Rexmont**	**0.22±0.01^a^**	**0.23±0.04^a^**	**0.14±0.00^a^**
**16**	**Tima**	**1.48±0.12^a^**	**0.81±0.07^b^**	**0.76±0.08^b^**
**17**	**Hong Cheuh Zai**	**1.39±0.16^a^**	**0.64±0.05^b^**	**0.60±0.05^b^**
**18**	**IR 8**	**2.22±0.12^a^**	**1.44±0.10^b^**	**0.97±0.09^c^**
**19**	**NERICA18**	**0.55±0.03^a^**	**0.36±0.03^b^**	**0.12±0.04^c^**
**20**	**Salinas9**	**0.73±0.04^a^**	**0.66±0.02^ab^**	**0.46±0.11^b^**
**21**	**IR29**	**0.93±0.12^a^**	**0.64±0.15^a^**	**0.61±0.09^a^**
**22**	**FL478**	**3.86±0.50^a^**	**1.78±0.19^b^**	**1.58±0.05^b^**
**23**	**Gaisen-Mochi**	**2.02±0.22^a^**	**0.98±0.08^b^**	**0.66±0.08^b^**
**24**	**Senshou**	**0.57±0.04^a^**	**0.33±0.02^b^**	**0.24±0.04^b^**
**25**	**Iruma-Nishiki**	**1.32±0.11^a^**	**0.63±0.04^b^**	**0.75±0.05^b^**
**26**	**Okka-Modoshi**	**1.02±0.11^a^**	**0.37±0.04^b^**	**0.36±0.08^b^**
**27**	**Bouzu-Mochi**	**0.56±0.05^a^**	**0.39±0.08^ab^**	**0.28±0.04^b^**
**28**	**Akage**	**1.10±0.12^a^**	**0.70±0.12^b^**	**0.51±0.04^b^**
**29**	**Hassokuho**	**1.15±0.14^a^**	**0.40±0.05^b^**	**0.35±0.03^b^**
**30**	**Kyoutoasahi**	**1.77±0.17^a^**	**1.03±0.05^b^**	**0.60±0.15^b^**
**31**	**Akamai**	**1.72±0.13^a^**	**0.74±0.08^b^**	**0.57±0.01^b^**
**32**	**Karahoushi**	**2.20±0.10^a^**	**0.88±0.05^b^**	**0.46±0.04^c^**
**33**	**Waisei-Murasaki-Ine**	**0.51±0.03^a^**	**0.31±0.07^b^**	**0.16±0.03^b^**
**34**	**Musashino-Murasaki**	**0.85±0.07^a^**	**0.27±0.06^b^**	**0.13±0.02^b^**

**Table 4:** SPAD value of the third leaf blade under control, SA-pH 8.3 (pH 8.3: 50 mM NaHCO_3_), and SA-pH 8.7 (pH 8.7: 47.5 mM NaHCO_3_ + 1.25 mM Na_2_CO_3_) conditions. Data represent the means ± SE of four replicates. Different alphabets accompanying each value indicate statistically significant differences among treatments, as determined by one-way ANOVA followed by Tukey’s test (*p* < 0.05).

No.	Name	SPAD		
		Control	pH 8.3	pH 8.7
**1**	**Naba**	**38.48 ± 0.51^a^**	**33.60±0.88^a^**	**15.10±4.08^b^**
**2**	**Sh**we **Nang Gyi**	**38.40 ± 0.76^a^**	**34.43±0.74^a^**	**23.03±3.18^b^**
**3**	**Pinulupot1**	**40.20 ± 0.62^a^**	**35.90±1.17^b^**	**35.38±0.81^b^**
**4**	**Muha**	**38.28 ± 1.88^a^**	**37.90±0.99^a^**	**34.15±2.95^a^**
**5**	**Nepal8**	**34.53 ± 0.48^a^**	**33.28±0.93^a^**	**27.95±2.06^b^**
**6**	**Jarjan**	**33.75 ± 0.52^a^**	**35.63±0.78^a^**	**34.80±1.09^a^**
**7**	**Kalo Dhan**	**32.05 ± 1.18^a^**	**35.33±1.32^a^**	**34.38±1.85^a^**
**8**	**Anjana Dhan**	**32.78 ± 0.34^a^**	**33.60±1.19^a^**	**30.40±1.36^a^**
**9**	**ARC7291**	**33.78 ± 0.59^a^**	**29.38±1.16^b^**	**30.88±0.34^ab^**
**10**	**Nepal555**	**32.88 ± 0.32^a^**	**31.03±2.19^a^**	**35.40±1.39^a^**
**11**	**Basilanon**	**35.50 ± 0.38^b^**	**39.18±0.98^a^**	**37.03±0.63^ab^**
**12**	**Jaguary**	**38.85 ± 0.44^a^**	**38.08±1.20^a^**	**38.65±0.79^a^**
**13**	**Khau Mac Kho**	**36.63 ± 1.24^a^**	**38.13±1.67^a^**	**33.60±1.71^a^**
**14**	**Padi Perak**	**39.58 ± 1.23^a^**	**39.10±1.55^a^**	**35.15±2.75^a^**
**15**	**Rexmont**	**35.53 ± 0.81^a^**	**42.50±1.33^a^**	**36.23±4.20^a^**
**16**	**Tima**	**39.90 ± 0.48^a^**	**38.23±2.82^a^**	**40.95±0.90^a^**
**17**	**Hong Cheuh Zai**	**36.58 ± 1.12^a^**	**37.45±1.58^a^**	**37.65±1.29^a^**
**18**	**IR8**	**39.23 ± 1.32^a^**	**38.88±0.61^a^**	**35.65±2.48^a^**
**19**	**NERICA18**	**34.95 ± 0.65^a^**	**36.23±0.67^a^**	**11.98±4.38^b^**
**20**	**Salinas9**	**37.53 ± 0.21^a^**	**36.53±0.40^a^**	**37.95±1.67^a^**
**21**	**IR29**	**36.30 ± 0.62^a^**	**37.20±0.40^a^**	**30.03±3.93^a^**
**22**	**FL478**	**42.00 ± 2.04^a^**	**42.83±0.37^a^**	**40.58±0.98^a^**
**23**	**Gaisen-Mochi**	**37.75 ± 1.79^a^**	**41.65±0.52^a^**	**27.68±3.73^b^**
**24**	**Senshou**	**37.53 ± 0.56^a^**	**36.48±1.30^a^**	**28.05±1.95^b^**
**25**	**Iruma-Nishiki**	**41.35 ± 0.55^a^**	**43.58±0.43^a^**	**41.70±0.91^a^**
**26**	**Okka-Modoshi**	**33.50 ± 0.20^a^**	**29.30±1.72^a^**	**28.75±1.76^a^**
**27**	**Bouzu-Mochi**	**35.60 ± 1.03^a^**	**34.48±1.80^a^**	**29.40±3.34^a^**
**28**	**Akage**	**38.15 ± 0.50^a^**	**39.03±0.88^a^**	**28.93±4.74^a^**
**29**	**Hassokuho**	**37.68 ± 0.51^a^**	**34.10±1.59^a^**	**24.65±0.76^b^**
**30**	**Kyoutoasahi**	**41.08 ± 0.41^a^**	**34.75±2.44^ab^**	**33.73±1.59^b^**
**31**	**Akamai**	**37.40 ± 1.58^a^**	**32.05±1.38^a^**	**32.05±1.48^a^**
**32**	**Karahoushi**	**35.95 ± 1.70^a^**	**31.50±0.97^a^**	**36.98±1.54^a^**
**33**	**Waisei-Murasaki-Ine**	**40.15 ± 0.49^a^**	**39.13±0.87^a^**	**23.03±2.93^b^**
**34**	**Musashino-Murasaki**	**36.78 ± 0.48^a^**	**18.53±3.78^b^**	**8.15±1.42^c^**

**Table 5:** Length of the roots and shoots under control and SA-pH 8.3 (pH 8.3: 50 mM NaHCO_3_) conditions. Data represent the means ± SE of four replicates. Asterisk accompanying the values indicates a statistically significant difference between treatments, as determined by F-tests and Student’s T-tests (*p* < 0.05).

No.	Name	Root length (cm)		Shoot length (cm)	
		Control	pH 8.3	Control	pH 8.3
**1**	**Naba**	**26.83±1.24**	20.88±1.18*	**48.38±0.81**	34.93±1.86*
**2**	**Shwe Nang Gyi**	**23.68±0.48**	18.88±0.61*	**55.23±0.84**	48.58±0.54*
**11**	**Basilanon**	**22.88±1.76**	**19.88±0.82**	**60.75±1.47**	46.88±0.70*
**12**	**Jaguary**	**36.00±1.05**	31.95±0.60*	**82.15±0.98**	64.63±1.31*
**15**	**Rexmont**	**28.45±0.81**	25.95±0.38*	**47.15±1.31**	33.80±0.54*
**20**	**Salinas9**	**21.53±0.51**	18.13±0.51*	**47.48±0.35**	42.10±0.55*
**21**	**IR29**	**18.53±1.00**	**18.20±0.46**	**51.93±0.32**	44.63±0.93*
**22**	**FL478**	**21.80±0.96**	15.60±0.56*	**63.95±1.01**	54.40±1.24*
**25**	**Iruma-Nishiki**	**25.30±0.39**	**22.10±0.35**	**75.50±1.47**	58.10±1.05*
**26**	**Okka-Modoshi**	**26.00±0.29**	21.58±0.35*	**71.73±0.94**	52.28±0.51*
**29**	**Hassokuho**	**23.38±1.06**	18.75±0.78*	**69.58±1.14**	55.18±0.86*
**35**	**Koshihikari**	**26.40±0.23**	20.60±0.14*	**61.90±1.12**	53.50±2.07*

**Table 6:** Fresh weight of the roots (RFW), leaf sheath (LSFW), and leaf blades (LBFW) under control and SA-pH 8.3 (pH 8.3: 50 mM NaHCO_3_) conditions. Data represent the means ± SE of four replicates. Asterisk accompanying each value indicates a statistically significant difference between treatments, as determined by F-tests and Student’s T-tests (*p* < 0.05).

No.	Name	RFW (g)		LSFW (g)		LBFW (g)	
		Control	pH 8.3	Control	pH 8.3	Control	pH 8.3
**1**	**Naba**	**2.70±0.12**	1.07±0.26*	**3.23±0.03**	1.29±0.33*	**1.80±0.02**	0.65±0.16*
**2**	**Sh**we **Nang Gyi**	**2.47±0.09**	**2.56±0.10**	**4.38±0.35**	3.19±0.33*	**2.40±0.25**	**1.79±0.16**
**11**	**Basilanon**	**1.64±0.28**	**1.26±0.13**	**2.21±0.39**	**1.21±0.09**	**1.21±0.25**	**0.66±0.04**
**12**	**Jaguary**	**6.83±0.31**	**5.13±0.68**	**12.51±0.40**	5.96±0.74*	**6.78±0.24**	3.46±0.47*
**15**	**Rexmont**	**1.68±0.20**	**1.34±0.17**	**2.54±0.32**	1.13±0.13*	**1.20±0.28**	**0.50±0.05**
**20**	**Salinas9**	**2.78±0.42**	**2.24±0.05**	**4.59±0.70**	**2.73±0.11**	**2.73±0.41**	**1.54±0.06**
**21**	**IR29**	**2.12±0.25**	**2.18±0.19**	**4.45±0.64**	**2.57±0.15**	**2.57±0.39**	1.32±0.14*
**22**	**FL478**	**3.44±0.34**	**3.23±0.14**	**5.89±0.67**	3.20±0.06*	**3.15±0.40**	1.83±0.10*
**25**	**Iruma-Nishiki**	**4.49±0.30**	**3.85±0.48**	**9.82±0.26**	6.27±0.81*	**6.24±0.34**	3.70±0.43*
**26**	**Okka-Modoshi**	**4.17±0.57**	1.82±0.08*	**7.22±0.81**	1.44±0.13*	**5.03±0.77**	0.92±0.07*
**29**	**Hassokuho**	**3.26±0.60**	**1.99±0.36**	**5.65±1.02**	2.30±0.14*	**3.38±0.73**	**1.24±0.20**
**35**	**Koshihikari**	**3.78±0.11**	2.13±0.09*	**6.40±0.22**	2.35±0.04*	**3.48±0.03**	1.21±0.03*

**Table 7:** Dry weight of the roots (RDW), leaf sheath (LSDW), and leaf blades (LBDW) under control and SA-pH 8.3 (pH 8.3: 50 mM NaHCO_3_) conditions. Data represent the means ± SE of four replicates. Asterisk accompanying each value indicates a statistically significant difference between treatments, as determined by F-tests and Student’s T-tests (*p* < 0.05).

No.	Name	RDW (g)		LSDW (g)		LBDW (g)	
		Control	pH 8.3	Control	pH 8.3	Control	pH 8.3
**1**	**Naba**	**0.26±0.02**	0.13±0.03*	**0.41±0.01**	**0.27±0.07**	**0.45±0.01**	0.22±0.05*
**2**	**Shwe Nang Gyi**	**0.30±0.01**	0.26±0.00*	**0.59±0.04**	**0.64±0.06**	**0.62±0.06**	**0.58±0.05**
**11**	**Basilanon**	**0.23±0.08**	**0.13±0.01**	**0.27±0.04**	**0.21±0.02**	**0.25±0.01**	**0.21±0.02**
**12**	**Jaguary**	**0.57±0.02**	**0.46±0.06**	**1.13±0.03**	**0.83±0.12**	**1.35±0.05**	0.87±0.12*
**15**	**Rexmont**	**0.18±0.02**	**0.13±0.02**	**0.29±0.02**	0.18±0.02*	**0.28±0.06**	**0.14±0.01**
**20**	**Salinas9**	**0.24±0.02**	**0.21±0.00**	**0.46±0.06**	**0.45±0.01**	**0.63±0.08**	**0.42±0.02**
**21**	**IR29**	**0.21±0.02**	**0.19±0.01**	**0.44±0.07**	**0.38±0.02**	**0.59±0.09**	**0.38±0.03**
**22**	**FL478**	**0.45±0.01**	**0.40±0.05**	**0.98±0.04**	**1.00±0.14**	**1.37±0.07**	0.99±0.13*
**25**	**Iruma-Nishiki**	**0.33±0.04**	**0.30±0.02**	**0.58±0.07**	**0.48±0.01**	**0.67±0.09**	**0.48±0.02**
**26**	**Okka-Modoshi**	**0.42±0.06**	0.18±0.02*	**0.73±0.07**	0.24±0.02*	**1.03±0.14**	0.26±0.01*
**29**	**Hassokuho**	**0.32±0.05**	**0.20±0.04**	**0.62±0.10**	**0.40±0.03**	**0.76±0.16**	**0.35±0.06**
**35**	**Koshihikari**	**0.37±0.01**	0.20±0.02*	**0.85±0.03**	0.46±0.02*	**0.88±0.01**	0.37±0.01*

**Table 8:** Chlorophyll a, b, and total chlorophyll concentrations of the third leaf blades under control and SA-pH 8.3 (pH 8.3: 50 mM NaHCO_3_) conditions. Data represent the means ± SE of four replicates. Asterisk accompanying each value indicates a statistically significant difference between treatments, as determined by F-tests and Student’s T-tests (*p* < 0.05).

No.	Name	Chl-a (µmol/g FW)		Chl-b (µmol/g FW)		Total chl (µmol/g FW)	
		Control	pH 8.3	Control	pH 8.3	Control	pH 8.3
**1**	**Naba**	**2.53±0.07**	**2.42±0.22**	**0.57±0.02**	**0.50±0.05**	**3.11±0.09**	**2.92±0.27**
**2**	**Shwe Nang Gyi**	**2.58±0.05**	**2.28±0.12**	**0.62±0.02**	**0.53±0.03**	**3.20±0.07**	**2.81±0.15**
**11**	**Basilanon**	**2.48±0.30**	**1.80±0.13**	**0.61±0.06**	**0.44±0.05**	**3.08±0.36**	**2.24±0.17**
**12**	**Jaguary**	**1.81±0.08**	**1.99±0.05**	**0.44±0.02**	**0.43±0.02**	**2.25±0.10**	**2.42±0.07**
**15**	**Rexmont**	**2.35±0.05**	**2.45±0.03**	**0.61±0.01**	**0.57±0.02**	**2.97±0.06**	**3.02±0.04**
**20**	**Salinas9**	**2.27±0.08**	**2.55±0.20**	**0.64±0.03**	**0.63±0.06**	**2.91±0.10**	**3.17±0.26**
**21**	**IR29**	**2.33±0.07**	**2.45±0.15**	**0.61±0.02**	**0.58±0.04**	**2.94±0.08**	**3.03±0.19**
**22**	**FL478**	**2.59±0.04**	2.93±0.05*	**0.68±0.02**	**0.76±0.02**	**3.27±0.07**	3.69±0.07*
**25**	**Iruma-Nishiki**	**2.00±0.13**	**2.15±0.04**	**0.47±0.03**	**0.47±0.01**	**2.47±0.16**	**2.62±0.04**
**26**	**Okka-Modoshi**	**2.18±0.11**	**1.68±0.28**	**0.56±0.04**	0.36±0.06*	**2.75±0.15**	**2.04±0.34**
**29**	**Hassokuho**	**1.89±0.10**	**2.15±0.31**	**0.43±0.03**	**0.48±0.08**	**2.32±0.12**	**2.63±0.39**
**35**	**Koshihikari**	**2.24±0.19**	**1.95±0.26**	**0.65±0.08**	0.39±0.05*	**2.89±0.14**	**2.34±0.31**

## 4 Experimental Design, Materials and Methods

### 4.1 Cultivation and sampling

The used plant materials are listed in Table 1. The rice seedlings were cultivated in a greenhouse at Hiroshima University, Japan. Rice seeds were wrapped in paper towels and bathed at 60 °C for 10 min, then soaked in 0.1 % fungicide for 2 days. After surface sterilization, the seeds were placed in a net-floating hydroponic solution containing half-strength Kimura B solution [2]. The pH was adjusted to 5.5 using 2 N HCl or 2 N NaOH every day. Four-week-old rice seedlings were exposed to control conditions (0 mM Na^+^, pH 5.5), and two kinds of saline-alkaline treatment (SA- pH 8.3, pH 8.0–8.3, 50 mM NaHCO_3_; SA-pH 8.7: pH 8.5–8.7, 47.5 mM NaHCO_3_ + 1.25 mM Na_2_CO_3_) for the first screening, or one saline-alkaline treatment (pH 8.0–8.3, 50 mM NaHCO_3_) for the second screening, respectively. The rice seedlings were treated for each stress during 2 weeks. At the time of sampling, the SPAD value was measured on the third leaf below the top fully expanded leaf. Afterward, the rice plants were carefully washed three times each with tap water and then with deionized water using a bucket. The plants were then separated into roots, LS, and LB. The samples were sealed in envelopes and dried at 70 °C.

### 4.2 Chlorophyll concentrations

For chlorophyll extraction, about 0.2 g of the fresh third leaf blades below the topmost fully expanded leaf blades on the main stem were collected and soaked into 10 mL of dimethylformamide (DMF) at 4 °C for 3 days in dark. The extracts were diluted 10 times, and their absorbance at 633.8 and 646.8 nm was measured using a spectrophotometer (UV-1850, SHIMADZU, Kyoto, Japan). The chlorophyll concentrations were calculated as previously described [9].

### 4.3 Statistical analysis

Statistical analyses were performed in R version 4.0.3. One-way analysis of variance (ANOVA) followed by the Tukey–Kramer test was used for multiple group comparisons. For comparisons between two groups, F-tests and Student’s T-tests were conducted.

## Limitations

The experiment was conducted under natural sunlight in a greenhouse. Therefore, reproducibility of the dataset may be limited in regions with different environmental conditions. Environmental differences should be considered when replicating the experiment in other locations.

## Ethics Statement

The authors confirm that they have read and follow the ethical requirements for publication in Data in Brief. The current work does not involve human subjects, animal experiments, or any data collected from social media platforms.

## CRediT Author Statement

**Mami Nampei:** Conceptualization, Methodology, Formal analysis, Investigation, Visualization, Writing – original draft, Writing – Review & Editing, Resources, Funding acquisition. **Akihiro Ueda:** Conceptualization, Writing – Review & Editing, Funding acquisition, Resources, Supervision.

## Data Availability

Mendeley DataGrowth profiling in screening of rice with saline-alkaline tolerance (Original data).
